# Sex-specific role of education on the associations of socioeconomic status indicators with obesity risk: A population-based study in South Korea

**DOI:** 10.1371/journal.pone.0190499

**Published:** 2018-01-03

**Authors:** Woojin Chung, Jaeyeun Kim, Seung-ji Lim, Sunmi Lee

**Affiliations:** 1 Department of Health Policy, Graduate School of Public Health, Yonsei University, Seoul, Republic of Korea; 2 Institute of Health Services Research, Yonsei University, Seoul, Republic of Korea; 3 Department of Public Health, Graduate School, Yonsei University, Seoul, Republic of Korea; 4 Health Insurance Policy Research Institute, National Health Insurance Service, Wonju-si, Republic of Korea; Tongji Med College, HUST, CHINA

## Abstract

**Background:**

No study of obesity risk for people in developed countries has conducted a multi-dimensional analysis of the association of socioeconomic status with obesity. In this paper, we investigated if education functions as either a confounder or an effect modifier in the association of another socioeconomic status indicator with obesity.

**Methods:**

This cross-sectional study analyzed data of an adult population sample (10,905 men and 14,580 women) from the Korea National Health and Nutrition Examination Survey (2010–2014). The study performed multivariate logistic regression analyses for three education levels and four indicators of socioeconomic status (i.e., marital status, residential area, occupation, and income).

**Results:**

The overall prevalence of obesity was 38.1% in men and 29.1% in women (*p* < 0.001). In men, while education functioned as an effect modifier in the association between marital status and obesity (*p* for interaction = 0.006), it functioned as both a confounder (*p* < 0.001) and an effect modifier (*p* for interaction < 0.001) in the association between residential area and obesity. In contrast, in women, education functioned as a confounder in the association of residential area with obesity (*p* = 0.010). However, it functioned as both a confounder (*p* < 0.001) and an effect modifier (*p* for interaction = 0.012) in the association between income and obesity. A prediction showed that unlike in women, education was positively associated with obesity risk for some socioeconomic indicator groups in men; for example, in a rural resident group, a higher level of education increased the probability of being obese by 19.7%.

**Conclusions:**

The present study suggests the need to examine sex-specific studies regarding the role of education on the association between other socioeconomic status indicators and obesity. This should be considered in planning education policies to reduce the risk of obesity.

## Introduction

Worldwide, obesity has become an important public health problem. Obesity can cause various diseases and a diminished quality of life for individuals [1], and it can cause a heavy economic burden by increasing a society’s medical expenditures, decreasing manpower, and thereby reducing labor productivity [2,3].

Regardless of whether they were for academic curiosity or policy development, numerous studies have examined factors associated with obesity risk. Among these factors, the association between socioeconomic status and obesity risk has attracted much attention across many disciplines. Generally, the consensus has been that in developed countries, a higher socioeconomic status is associated with a lower risk of obesity in both men and women [4–6].

Meanwhile, recent studies from developed countries point to a more complex association between socioeconomic status indicators and obesity risk, thereby asking for more and better research on the association. For example, studies from Canada [7], France [8], Luxembourg [9], the United States [10], and South Korea [11] suggest that the association between a particular socioeconomic status indicator and obesity risk may not only be positive in one sex, but negative in the other sex.

Unfortunately, despite a lot of attention paid to the associations between socioeconomic status indicators and obesity risk, a study of a multi-dimensional analysis on the associations has not been performed for people in developed countries. This lack of studies may lead to the unavailability of adequate information for developing theories and designing efficient public health policies aimed at reducing obesity risk in specific groups of people.

Therefore, the aim of the present study was to employ a multi-dimensional analysis and examine the role of education on the association between other socioeconomic status indicators (such as marital status, residential area, occupation, and income) and obesity. In the present study, we elected to focus on the role of education among the various socioeconomic status indicators because education level is established during early adulthood and generally remains unchanged unlike the other socioeconomic indicators that are more susceptible to change.

In this study, we sought to identify education as either a confounder or an effect modifier or both a confounder and effect modifier in the association between another socioeconomic status indicator and obesity. In addition, after considering the role of education in the association between another socioeconomic status indicator and obesity, the study aimed to investigate if a higher level of education was associated with a reduced risk of obesity in both men and women. To fulfill the aims of the study, we analyzed a sample adult population aged ≥25 years from the nationally representative data in South Korea; this population was selected because the country is one of the largest developed countries in the world [12], and people in this age group were thought to have most likely completed their education.

## Materials and methods

### Data source and study sample

We used data from the Fifth and Sixth Korea National Health and Nutrition Examination Survey (KNHANES V and VI, 2010–2014), performed by the Korea Centers for Disease Control and Prevention. The sampling design for the KNHANES was a stratified, multistage probability survey of the non-institutionalized general population of South Korea. This survey included a health interview, health examination, and a nutrition survey that were conducted at participants’ homes, as well as a physical examination that was conducted by physicians at designated examination centers.

For KNHANES V and VI, 41,102 individuals participated in the interviews (8,958 in 2010; 8,518 in 2011; 8,058 in 2012; 8,018 in 2013; and 7,550 in 2014). This study chose 29,266 participants from the total number of participants in the 2010–2014 survey, including only those aged ≥25 years (n = 29,752) to ensure they had completed their education [13]. As bodyweight of pregnant or breast-feeding (n = 486) women is affected by childbearing, they were excluded from the study.

Finally, the study analyzed the findings from the 25,485 (87.1%) participants (10,905 men and 14,580 women) with complete information. The χ^2^ tests showed no significant differences in participant characteristics before and after the exclusion of participants with incomplete information (for age, *p*-values were 0.485 in men and 0.185 in women; for residential area, *p*-values were 0.507 in men and 0.271 in women).

All KNHANES participants provided written consent to participate in the survey and for their personal data to be used. This study used publicly available data, and ethical approval was obtained from the institutional review board of Yonsei University Graduate School of Public Health (IRB No. 2-1040939-AB-N-01-2016-157).

### Measures and variables

The obesity status of each participant was determined anthropometrically using data from the physical examination. As recommended by Asian criteria suggested by the World Health Organization, general obesity was defined as a body mass index of ≥25 kg/m^2^ [14].

This study examined five socioeconomic status indicators: education, marital status, residential area, occupation, and income. Education, defined as the highest level of formal education completed at the time of interview, was divided into the following three levels: middle school or less, high school, and college or higher. Marital status was denoted as married or non-married (i.e., never married, separated, widowed, or divorced). Residential area was denoted as urban area or rural area. Occupation was defined according to the following three groups: office worker, manual worker, and no job (e.g., those who had no job in the labor market). For income, this study calculated an equivalized monthly household income for each household to adjust for household size ([monthly overall household income] [household size]^-0.5^) [15,16] that divided participants into four quartiles.

Nine variables were used in this study as potential confounders, and except survey year, these variables were grouped into the following two categories: sex (men and women), smoking status (smoking and non-smoking), risk from alcohol intake (no or low risk and medium or higher risk), routine walk exercise activity (active and inactive), daily sleep duration (short sleep and long sleep), daily energy intake (under-reported and not under-reported), self-perceived stress level(stressed and not stressed), chronic disease (yes and no), and survey year.

In details, risk from alcohol intake was based on the sex-specific guidelines of the World Health Organization [17]. Routine walk exercise activity was categorized as “active” if a participant walked for at least 30 minutes per day, for ≥5 days per week [18]. Daily sleep duration was denoted as “short sleep” if a participant slept for ≤6 hours per day [19]. Daily energy intake obtained from a 24-hour dietary recall of a participant was defined as “under-reported” if the participant consumed energy lower than the participant’s estimated energy requirement (EER). The Institute of Medicine developed the EER predictive equations, where an individual’s EER is defined as the individual’s dietary energy intake required to maintain energy balance according to the individual’s age, sex, weight, height, and level of physical activity [20]. Chronic disease was defined as “yes” if a participant had at least one of the following diseases at the time of the survey: hypertension, dyslipidemia, and diabetes mellitus.

In a preliminary analysis, this study included age as a discrete variable and housing tenure as a proxy of wealth. However, because of the lack of significance and a high level of multicollinearity, this study posited age as a continuous variable and removed the housing tenure variable. For each multivariate model that focused on the role of education level on the association between each socioeconomic status indicator and obesity, the other socioeconomic status indicators were added as potential confounders to the above-mentioned potential confounders.

### Statistical analysis

We first tested differences in the distributions of variables among men and women using the *t*-test for continuous, age variable and the χ^2^ test for categorical variables. Second, the prevalence of obesity for each group of all socioeconomic status indicators was estimated and compared according to education level among men and women using χ^2^ tests. Third, we carried out a Wald test for the significance of the three-way interaction-effect term among sex, education and each socioeconomic status indicator in the logistic regression model with (1) three main-effect terms of sex, education and the socioeconomic status indicator, (2) the two-way interaction-effect term between education and the socioeconomic status indicator, and (3) the three-way interaction-effect term among sex, education and each socioeconomic status indicator. Because the three-way interaction-effect term was highly significant for every socioeconomic status indicator (*p* for interaction <0.0001), we stratified the remaining analyses by sex.

Fourth, to examine the role of education in the association between each socioeconomic status indicator and obesity, we employed three different methods as follows.

Method 1: To examine a possibility that education level modifies the association between each socioeconomic status indicator and obesity, we estimated the adjusted odds ratios (ORs) of obesity (and their 95% confidence intervals, CIs) for each socioeconomic status indicator with and without being stratified by education level for each sex; these were obtained from the logistic regression models adjusted for all the studied confounders. According to statistical rules of thumb distinguishing a confounder from an effect modifier, if the ORs of obesity for a socioeconomic status indicator without being stratified by education level were outside the range of the stratum-specific ORs of obesity for the socioeconomic status indicator, we considered that education was very likely a confounder. Meanwhile, if the ORs of obesity for a socioeconomic status indicator, not stratified by education level, were inside the range of the stratum-specific ORs of obesity for the socioeconomic status indicator and the stratum-specific ORs of obesity for that indicator were very different from one another, we postulated education very likely to be an effect modifier.

Method 2: To examine the role of education between each socioeconomic status indicator and obesity, we obtained a first set of unadjusted ORs of obesity for the socioeconomic status indicator in a logistic regression model with only the socioeconomic status indicator as an independent variable (Model 1). We then obtained the second set of unadjusted ORs of obesity for the socioeconomic status indicator in another logistic regression model (Model 2), after adding the main-effect term of education as well as an interaction-effect term between the socioeconomic status indicator and education to Model 1. If the first set of unadjusted ORs of obesity for the socioeconomic status indicator, obtained from Model 1, was significantly different from the second set, obtained from Model 2, based on the seemingly unrelated estimation method and Wald test [21], we considered education as a confounder, because the adding it significantly changed the association between the socioeconomic status indicator and obesity.

In addition, if the interaction-effect terms from combinations of the socioeconomic status indicator groups and education levels in relation to obesity in Models 2 were jointly significant, we considered education as an effect modifier, because the association between the socioeconomic status indicator and obesity changed significantly across education levels. As for marital status, for example, we have two combinations of marital status groups and education levels constructing the interaction-effect terms in relation to obesity; one is non-married and high school and the other non-married and college or higher. For the test for the joint significance of interaction-effect terms from two combinations of marital status groups and education levels, the null hypothesis states that ORs of interaction-effect terms from both combinations are set as zero, whereas the alternative hypothesis states that at least one OR of interaction-effect terms from both combinations is non-zero.

Method 3: To examine if the role of education between each socioeconomic status indicator and obesity changes from the previous, unadjusted models to adjusted models, we conducted additional analyses of the models in Method 2 using all studied confounders and the other socioeconomic status indicators. In the case of these adjusted models, this study found no evidence of lack of goodness-of-fit in each model; *p*-values based on the Hosmer–Lemeshow statistic were ≥ 0.178.

Finally, to examine if obesity risk decreases with education after considering the role of education in the association between each socioeconomic indicator and obesity, we estimated the change in an individual’s predicted probability of being obese (and its 95% CIs), if the individual belonging to a socioeconomic status indicator group would increase the individual’s level of education from the lowest level (middle school or less) to a higher level (either high school; or college or higher), all the other factors held constant at the individual’s own values.

This study used the STATA version 13 (StataCorp, College Station, TX, USA) and conducted all analyses and tests using the method to deal with the complex survey design, that is, using the weighted sample. However, for convenience, the descriptive statistics in Table 1 were shown as unweighted; *p*-values < 0.05 were regarded as statistically significant.

**Table 1:** General characteristics of the study sample by sex and education level.

Characteristics	Men (N = 10905)				Women (N = 14580)				
	Middle school or less	High school	College or higher	*p*	Middle school or less	High school	College or higher		*p*
Age in years (mean)	64.6	50.3	44.5	<0.001	64.7	46.6	39.4		<0.001
Non-married	12.0	17.6	20.3	<0.001	36.4	15.9	25.1		<0.001
Rural	35.0	20.6	11.4	<0.001	30.8	15.0	9.0		<0.001
Occupation				<0.001					<0.001
No job	37.1	21.9	15.5		58.0	49.2	45.2		
Office worker	2.5	18.2	58.0		1.2	14.0	43.3		
Manual worker	60.4	59.9	26.5		40.8	36.8	11.5		
Income, quartiles				<0.001					<0.001
Lowest	45.4	17.6	8.0		47.8	13.6	6.0		
2nd lowest	27.4	29.7	20.3		24.6	29.3	20.5		
3rd lowest	16.9	28.3	31.8		15.7	29.5	31.7		
Highest	10.3	24.4	39.9		11.9	27.6	41.8		
Smoking	35.5	45.3	40.5	<0.001	4.6	7.1	4.0		<0.001
Medium or higher risk from alcohol intake	31.5	51.9	55.8	<0.001	14.8	33.2	33.0		<0.001
Active, walk exercise	37.9	40.5	40.8	<0.001	33.0	36.9	35.3		<0.001
Short sleep	42.7	41.4	43.5	0.295	52.8	40.3	34.6		<0.001
Under-reporting of energy intake	31.3	29.1	30.4	0.435	20.9	21.2	18.6		0.004
Stressful	16.1	21.6	27.7	<0.001	26.4	25.6	29.0		0.004
Chronic disease	41.5	25.8	15.9	<0.001	49.0	16.2	5.3		<0.001
Survey year				0.335					0.054
2010	23.4	22.2	22.5		22.1	21.5	20.9		
2011	22.1	21.9	21.2		22.3	21.4	20.3		
2012	19.5	19.9	19.2		20.6	19.9	19.4		
2013	17.6	19.6	18.9		18.1	19.7	20.1		
2014	17.4	16.5	18.1		16.9	17.5	19.3		
All participants	30.6	32.6	36.8		44.5	29.7	25.9		

For the sake of brevity, the descriptive statistics were shown as % and unweighted.

*P*-values were obtained by the χ2 test considering the complex survey design.

For age, *P*-values were obtained by the ANOVA test considering the complex survey design.

*N* number

## Results

In Table 1, the participant characteristics that were significantly different across education levels for each sex, with the exception of sleep duration (*p* = 0.295), energy intake (*p* = 0.435), and survey year (*p* = 0.335) in men and survey year (*p* = 0.054) in women are shown.

The percentage of obesity was estimated in 38.1% (standard error, 0.6) in men and 29.1% (standard error, 0.5) in women, being significantly different between sexes (*p* < 0.001). The percentage of obesity in each socioeconomic status indicator group according to educational level and sex is shown in Table 2. The obesity rate for each group of socioeconomic status indicators varied significantly across education levels except for office worker (*p* = 0.449), the second lowest income group (*p* = 0.122), the third lowest income group (*p* = 0.699), and the highest income group (*p* = 0.132) in men. This suggests that each education level may play a differentiated role on the association between a socioeconomic status indicator and obesity in either men or women.

**Table 2:** Percentage of obesity in each socioeconomic status indicator group by sex and educational level.

Characteristics	Men (N = 10905)							Women (N = 14580)							
	Middle school or less		High school		College or higher		*p*	Middle school or less		High school		College or higher		*p*	
	Rate	(SE)	Rate	(SE)	Rate	(SE)		Rate	(SE)	Rate	(SE)	Rate	(SE)		
All participants	32.3	(1.0)	39.1	(1.0)	40.7	(1.0)	<0.001	39.8	(0.8)	28.0	(0.8)	16.0	(0.7)	<0.001	
Marital status															
Married	32.6	(1.1)	38.6	(1.1)	43.1	(1.1)	<0.001	40.9	(0.9)	28.6	(0.9)	16.9	(0.9)	<0.001	
Non-married	30.2	(2.9)	40.9	(2.2)	34.2	(2.0)	0.011	37.7	(1.3)	25.0	(1.9)	13.7	(1.4)	<0.001	
Residential area							<0.001^a^								
Urban	35.4	(1.2)	38.7	(1.1)	39.9	(1.0)	0.029	39.4	(0.9)	26.8	(0.9)	15.7	(0.8)	<0.001	
Rural	25.8	(1.5)	40.9	(2.3)	46.4	(3.0)	<0.001	40.7	(1.3)	33.8	(2.2)	18.8	(2.4)	<0.001	
Occupation															
No job	27.0	(1.5)	36.1	(2.3)	34.8	(2.6)	0.004	40.5	(0.9)	28.5	(1.2)	16.6	(1.1)	<0.001	
Office worker	48.5	(7.0)	44.3	(2.3)	41.9	(1.2)	0.449	31.7	(5.9)	25.6	(2.1)	14.6	(1.0)	<0.001	
Manual worker	34.1	(1.4)	38.4	(1.2)	40.8	(1.7)	0.007	39.1	(1.2)	28.2	(1.4)	19.2	(2.3)	<0.001	
Income, quartiles															
Lowest	26.5	(1.4)	36.5	(2.5)	38.9	(3.4)	<0.001	40.1	(1.0)	29.1	(2.2)	25.5	(3.7)	<0.001	
2nd lowest	35.8	(1.9)	40.5	(1.8)	41.5	(2.1)	0.122	40.1	(1.6)	31.6	(1.6)	19.7	(1.7)	<0.001	
3rd lowest	36.5	(2.4)	38.8	(1.8)	39.0	(1.7)	0.699	41.7	(1.8)	27.3	(1.6)	16.3	(1.2)	<0.001	
Highest	35.2	(3.1)	39.4	(2.0)	42.1	(1.5)	0.132	35.5	(2.2)	23.8	(1.5)	12.0	(1.0)	<0.001	

All analyses were conducted considering the complex survey design.

*P*-values were obtained by the χ2 test.

*N* number, *SE* standard error, *Obesity* body mass index ≥25

Table 3 displays the adjusted odds ratios (ORs) of obesity (and their 95% CIs) that were obtained from the logistic regression models adjusted for all the studied confounders for each socioeconomic status indicator with and without being stratified by education level for each sex. According to the statistical rules of thumb that help distinguish a confounder from an effect modifier described as Method 1 in the statistical analysis section, in men, education was very likely an effect modifier in the association between each socioeconomic status indicator and obesity. In contrast, in women, education was very likely to play a different role in the association between a socioeconomic status indicator and obesity, depending on which socioeconomic status indicator was associated with obesity; a confounder for both marital status and residential area; an effect modifier for income; and both a confounder and an effect modifier for occupation. Accordingly, the results from the statistical rules of thumb suggest that education may be either a confounder or an effect modifier or both in the association between a socioeconomic status indicator and obesity, being different between sexes.

**Table 3:** Adjusted odds ratios (and their 95% CIs) of obesity according to socioeconomic status indicators with and without being stratified by education level for each sex.

Characteristics	Men								Women							
	All		Middle school or less		High school		College or higher		All		Middle school or less		High school		College or higher	
	OR	(95% CI)	OR	(95% CI)	OR	(95% CI)	OR	(95% CI)	OR	(95% CI)	OR	(95% CI)	OR	(95% CI)	OR	(95% CI)
Marital status																
Married	1.00		1.00		1.00		1.00		1.00		1.00		1.00		1.00	
Non-married	0.90	(0.79–1.02)	0.89	(0.67–0.19)	1.10	(0.90–1.35)	0.69	(0.56–0.83)	0.92	(0.83–1.02)	0.87	(0.77–0.99)	0.83	(0.67–1.03)	0.78	(0.61–1.01)
Residential area																
Urban	1.00		1.00		1.00		1.00		1.00		1.00		1.00		1.00	
Rural	0.92	(0.81–1.04)	0.64	(0.53–0.77)	1.10	(0.89–1.35)	1.31	(1.02–1.68)	1.47	(1.32–1.64)	1.06	(0.93–1.20)	1.39	(1.13–1.73)	1.24	(0.90–1.72)
Occupation																
No job	1.00		1.00		1.00		1.00		1.00		1.00		1.00		1.00	
Office worker	1.57	(1.37–1.80)	2.54	(1.44–4.50)	1.41	(1.08–1.83)	1.35	(1.05–1.73)	0.49	(0.43–0.56)	0.68	(0.40–1.17)	0.86	(0.68–1.10)	0.86	(0.68–1.08)
Manual worker	1.27	(1.12–1.45)	1.40	(1.15–1.70)	1.10	(0.89–1.36)	1.29	(0.99–1.69)	1.10	(1.00–1.20)	0.95	(0.84–1.06)	0.98	(0.83–1.17)	1.20	(0.87–1.65)
Income, quartiles																
Lowest	1.00		1.00		1.00		1.00		1.00		1.00		1.00		1.00	
2nd lowest	1.41	(1.22–1.63)	1.54	(1.24–1.91)	1.19	(0.92–1.54)	1.11	(0.80–1.55)	0.80	(0.71–0.90)	1.00	(0.87–1.16)	1.13	(0.88–1.45)	0.72	(0.47–1.10)
3rd lowest	1.35	(1.16–1.55)	1.59	(1.25–2.02)	1.10	(0.85–1.44)	1.00	(0.74–1.36)	0.64	(0.57–0.72)	1.07	(0.90–1.27)	0.92	(0.71–1.19)	0.57	(0.38–0.85)
Highest	1.46	(1.27–1.69)	1.51	(1.11–2.04)	1.13	(0.87–1.48)	1.14	(0.84–1.54)	0.45	(0.39–0.51)	0.82	(0.67–1.01)	0.76	(0.58–1.00)	0.40	(0.26–0.62)
N	10905		3339		3555		4011		14580		6483		4324		3773	

All analyses were conducted considering the complex survey design.

All estimates were obtained from the logistic regression models, adjusted for age, smoking, alcohol intake, walk exercise, sleep duration, daily energy intake, self-perceived stress, chronic disease, and survey year.

*N* number, *OR* odds ratio, *CI* confidence interval, *Obesity* body mass index ≥25

According to Method 2 described in the statistical analysis section, unadjusted results of the main and interaction effects of each socioeconomic status indicator and education on obesity in men and in women, respectively, is given in Tables 4 and 5. In men, among all studied socioeconomic status indicators, only residential area showed significant differences in ORs of obesity (*p* < 0.001) from the model including only the socioeconomic status indicator (Model 1) to the model considering the main effect and the interaction effect of the socioeconomic status indicator and education (Model 2). As for interaction-effect terms between groups of each socioeconomic status indicator and education levels in regards to obesity in Model 2, those between groups of marital status and education levels (*p* for interaction = 0.005) and those between groups of residential area and education levels (*p* for interaction < 0.001) were jointly significant, respectively. Meanwhile, in women, residential area (*p* < 0.001), occupation (*p* = 0.003) and income (*p* < 0.001) showed significant differences in ORs of obesity from Model 1 to Model 2. Only the interaction-effect terms between groups of income and education levels in Model 2 were jointly significant (*p* for interaction = 0.027).

**Table 4:** Unadjusted results of the main and interaction effects of each socioeconomic status indicator and education on obesity in men.

Characteristics	Model 1		Model 2							
			Socioeconomic status indicator		Middle school or less	High school		College or higher		*p* for interaction
	OR	(95% CI)	OR	(95% CI)	OR	OR	(95% CI)	OR	(95% CI)	
Marital status (Ref: Married)			0.971*		1.00	1.30	(1.13–1.48)	1.56	(1.37–1.78)	
Non-married	0.90	(0.79–1.02)	0.89	(0.67–1.19)		1.23	(0.87–1.75)	0.77	(0.54–1.09)	0.005
Residential area (Ref: Urban)			< .001*		1.00	1.15	(1.00–1.32)	1.21	(1.06–1.39)	
Rural	0.92	(0.81–1.04)	0.64	(0.53–0.77)		1.72	(1.30–2.28)	2.05	(1.51–2.79)	<0.001
Occupation (Ref: No job)			0.184*		1.00	1.53	(1.19–1.95)	1.45	(1.11–1.89)	
Office worker	1.57	(1.37–1.80)	2.54	(1.44–4.50)		0.55	(0.29–1.04)	0.53	(0.28–1.00)	0.096
Manual worker	1.27	(1.12–1.45)	1.40	(1.15–1.70)		0.79	(0.59–1.05)	0.92	(0.66–1.28)	
Income, quartiles (Ref: Lowest)			0.485*		1.00	1.59	(1.23–2.05)	1.76	(1.29–2.40)	
2nd lowest	1.41	(1.22–1.63)	1.54	(1.24–1.91)		0.77	(0.55–1.07)	0.72	(0.48–1.09)	0.260
3rd lowest	1.35	(1.16–1.55)	1.59	(1.25–2.02)		0.70	(0.48–1.00)	0.63	(0.43–0.93)	
Highest	1.46	(1.27–1.69)	1.51	(1.11–2.04)		0.75	(0.50–1.14)	0.76	(0.50–1.15)	

All analyses were conducted considering the complex survey design.

*P-*values for interaction were obtained by the Wald test.

*P-values were obtained by the Wald test to examine if estimates of odds ratios of all socioeconomic status indicator groups differ jointly between Models 1and 2 on the basis of the seemingly unrelated estimation method.

Model 1 included each socioeconomic status indicator only.

Model 2 included two main-effect terms of each socioeconomic status indicator and education as well as the interaction-effect term of the two variables.

All estimates were obtained from logistic regression models.

*OR* odds ratio, *CI* confidence interval, *Obesity* body mass index ≥25, *Ref* reference group

**Table 5:** Unadjusted results of the main and interaction effects of each socioeconomic status indicator and education on obesity in women.

Characteristics	Model 1		Model 2							
			Socioeconomic status indicator		Middle school or less	High school		College or higher		*p* for interaction
	OR	(95% CI)	OR	(95% CI)	OR	OR	(95% CI)	OR	(95% CI)	
Marital status (Ref: Married)			0.265*		1.00	0.58	(0.51–0.65)	0.29	(0.25–0.34)	
Non-married	0.92	(0.83–1.02)	0.87	(0.77–0.99)		0.95	(0.75–1.21)	0.90	(0.67–1.19)	0.735
Residential area (Ref: Urban)			<0.001*		1.00	0.56	(0.50–0.63)	0.29	(0.25–0.33)	
Rural	1.47	(1.32–1.64)	1.06	(0.93–1.20)		1.32	(1.02–1.71)	1.18	(0.83–1.66)	0.095
Occupation (Ref: No job)			0.003*		1.00	0.59	(0.51–0.67)	0.29	(0.25–0.35)	
Office worker	0.49	(0.43–0.56)	0.68	(0.40–1.17)		1.26	(0.70–2.30)	1.26	(0.70–2.27)	0.664
Manual worker	1.10	(1.00–1.20)	0.95	(0.84–1.06)		1.04	(0.85–1.28)	1.27	(0.90–1.79)	
Income, quartiles (Ref: Lowest)			<0.001*		1.00	0.61	(0.49–0.76)	0.51	(0.35–0.76)	
2nd lowest	0.80	(0.71–0.90)	1.00	(0.87–1.16)		1.12	(0.85–1.49)	0.71	(0.46–1.12)	0.027
3rd lowest	0.64	(0.57–0.72)	1.07	(0.90–1.27)		0.86	(0.64–1.16)	0.53	(0.34–0.82)	
Highest	0.45	(0.39–0.51)	0.82	(0.67–1.01)		0.93	(0.67–1.28)	0.49	(0.30–0.79)	

All analyses were conducted considering the complex survey design.

*P-*values for interaction were obtained by the Wald test.

*P-values were obtained by the Wald test to examine if estimates of odds ratios of all socioeconomic status indicator groups differ jointly between Models 1and 2 on the basis of the seemingly unrelated estimation method.

Model 1 included each socioeconomic status indicator only.

Model 2 included two main-effect terms of each socioeconomic status indicator and education as well as the interaction-effect term of the two variables.

All estimates were obtained from logistic regression models.

*OR* odds ratio, *CI* confidence interval, *Obesity* body mass index ≥25, *Ref* reference group

These results suggest that in men, education may play a role as an effect modifier in the association between marital status and obesity, while functioning as both a confounder and an effect modifier in the association between residential area and obesity. In contrast, in women, education may work as a confounder in the association of each of residential area and occupation with obesity, while functioning as both a confounder and an effect modifier in the association between income and obesity.

After adjustments for all studied confounders, based on Method 3 described in the statistical analysis section, the results of the main and interaction effects of each socioeconomic status indicator and education on obesity are presented in Table 6 for men and Table 7 for women. In Table 6, the differences in the associations between each socioeconomic status indicator and obesity between Model 1 and Model 2 were significant for residential area (*p* < 0.001), the interaction-effect terms between groups of marital status and education levels with regard to obesity (*p* for interaction = 0.006), and those between residential area and education levels (*p* for interaction < 0.001) in Model 2 were jointly significant.

**Table 6:** Adjusted results of the main and interaction effects of each socioeconomic status indicator and education on obesity in men.

Characteristics	Model 1		Model 2							
			Socioeconomic status indicator		Middle school or less	High school		College or higher		*p* for interaction
	OR	(95% CI)	OR	(95% CI)	OR	OR	(95% CI)	OR	(95% CI)	
Marital status (Ref: Married)			0.421*		1.00	1.08	(0.92–1.25)	1.19	(1.00–1.42)	
Non-married	0.75	(0.65–0.87)	0.84	(0.62–1.13)		1.13	(0.78–1.64)	0.72	(0.50–1.03)	0.006
Residential area (Ref: Urban)			<0.001*		1.00	0.97	(0.82–1.13)	0.94	(0.79–1.12)	
Rural	1.02	(0.90–1.16)	0.68	(0.55–0.83)		1.70	(1.26–2.28)	2.00	(1.46–2.74)	<0.001
Occupation (Ref: No job)			0.125*		1.00	1.35	(1.03–1.75)	1.27	(0.95–1.68)	
Office worker	1.30	(1.10–1.55)	2.13	(1.20–3.79)		0.59	(0.31–1.12)	0.58	(0.31–1.09)	0.243
Manual worker	1.17	(1.01–1.36)	1.34	(1.08–1.65)		0.79	(0.58–1.06)	0.89	(0.64–1.24)	
Income, quartiles (Ref: Lowest)			0.167*		1.00	1.36	(1.04–1.78)	1.48	(1.06–2.06)	
2nd lowest	1.19	(1.02–1.40)	1.37	(1.09–1.72)		0.80	(0.57–1.13)	0.71	(0.47–1.08)	0.285
3rd lowest	1.06	(0.90–1.24)	1.35	(1.04–1.75)		0.73	(0.50–1.06)	0.62	(0.41–0.93)	
Highest	1.14	(0.96–1.34)	1.33	(0.97–1.82)		0.73	(0.48–1.13)	0.72	(0.47–1.10)	

All analyses were conducted considering the complex survey design.

*P-*values for interaction were obtained by the Wald test.

*P-values were obtained by the Wald test to examine if estimates of odds ratios of all socioeconomic status indicator groups differ jointly between Models 1and 2 on the basis of the seemingly unrelated estimation method.

Model 1 included each socioeconomic status indicator only.

Model 2 included two main-effect terms of each socioeconomic status indicator and education as well as the interaction-effect term of the two variables.

All estimates were obtained from logistic regression models, adjusted for age, smoking, alcohol intake, walk exercise, sleep duration, daily energy intake, self-perceived stress, chronic disease, survey year, and the other socioeconomic indicators.

*OR* odds ratio, *CI* confidence interval, *Obesity* body mass index ≥25, *Ref* reference group

**Table 7:** Adjusted results of the main and interaction effects of each socioeconomic status indicator and education on obesity in women.

Characteristics	Model 1		Model 2							
			Socioeconomic status indicator		Middle school or less	High school		College or higher		*p* for interaction
	OR	(95% CI)	OR	(95% CI)	OR	OR	(95% CI)	OR	(95% CI)	
Marital status (Ref: Married)			0.618*		1.00	0.74	(0.65–0.86)	0.46	(0.38–0.56)	
Non-married	0.71	(0.64–0.80)	0.73	(0.63–0.84)		1.04	(0.81–1.33)	0.97	(0.72–1.32)	0.914
Residential area (Ref: Urban)			0.010*		1.00	0.71	(0.62–0.82)	0.45	(0.37–0.54)	
Rural	1.23	(1.10–1.37)	1.07	(0.94–1.23)		1.28	(0.98–1.68)	1.19	(0.82–1.71)	0.166
Occupation (Ref: No job)			0.239*		1.00	0.76	(0.64–0.90)	0.46	(0.37–0.57)	
Office worker	0.81	(0.69–0.95)	0.75	(0.43–1.31)		1.29	(0.70–2.37)	1.25	(0.69–2.26)	0.855
Manual worker	1.17	(1.05–1.29)	1.07	(0.94–1.23)		0.95	(0.76–1.17)	1.07	(0.75–1.54)	
Income, quartiles (Ref: Lowest)			<0.001*		1.00	0.81	(0.64–1.02)	0.82	(0.54–1.25)	
2nd lowest	1.08	(0.94–1.23)	1.09	(0.92–1.28)		1.04	(0.78–1.39)	0.63	(0.39–1.01)	0.012
3rd lowest	0.94	(0.81–1.08)	1.16	(0.97–1.38)		0.78	(0.57–1.06)	0.49	(0.31–0.77)	
Highest	0.70	(0.60–0.82)	0.93	(0.75–1.15)		0.84	(0.60–1.17)	0.43	(0.26–0.70)	

All analyses were conducted considering the complex survey design.

*P-*values for interaction were obtained by the Wald test.

*P-values were obtained by the Wald test to examine if estimates of odds ratios of all socioeconomic status indicator groups differ jointly between Models 1and 2 on the basis of the seemingly unrelated estimation method.

Model 1 included each socioeconomic status indicator only.

Model 2 included two main-effect terms of each socioeconomic status indicator and education as well as the interaction-effect term of the two variables.

All estimates were obtained from logistic regression models, adjusted for age, smoking, alcohol intake, walk exercise, sleep duration, daily energy intake, self-perceived stress, chronic disease, survey year, and the other socioeconomic indicators.

*OR* odds ratio, *CI* confidence interval, *Obesity* body mass index ≥25, *Ref* reference group

Meanwhile, in women, residential area (*p* = 0.010) and income (*p* <0.001) showed significant differences in ORs of obesity between Model 1 and Model 2. Similar to the unadjusted model in Table 5, only the interaction-effect terms between groups of income and education levels in Model 2 were jointly significant (*p* for interaction = 0.012).

According to these adjusted results, in men, education may work as an effect modifier in the association between marital status and obesity and as both a confounder and an effect modifier in the association between residential area and obesity. In contrast, in women, education may work as a confounder in the association of residential area with obesity and as both a confounder and an effect modifier in the association between income and obesity.

**Fig 1:**
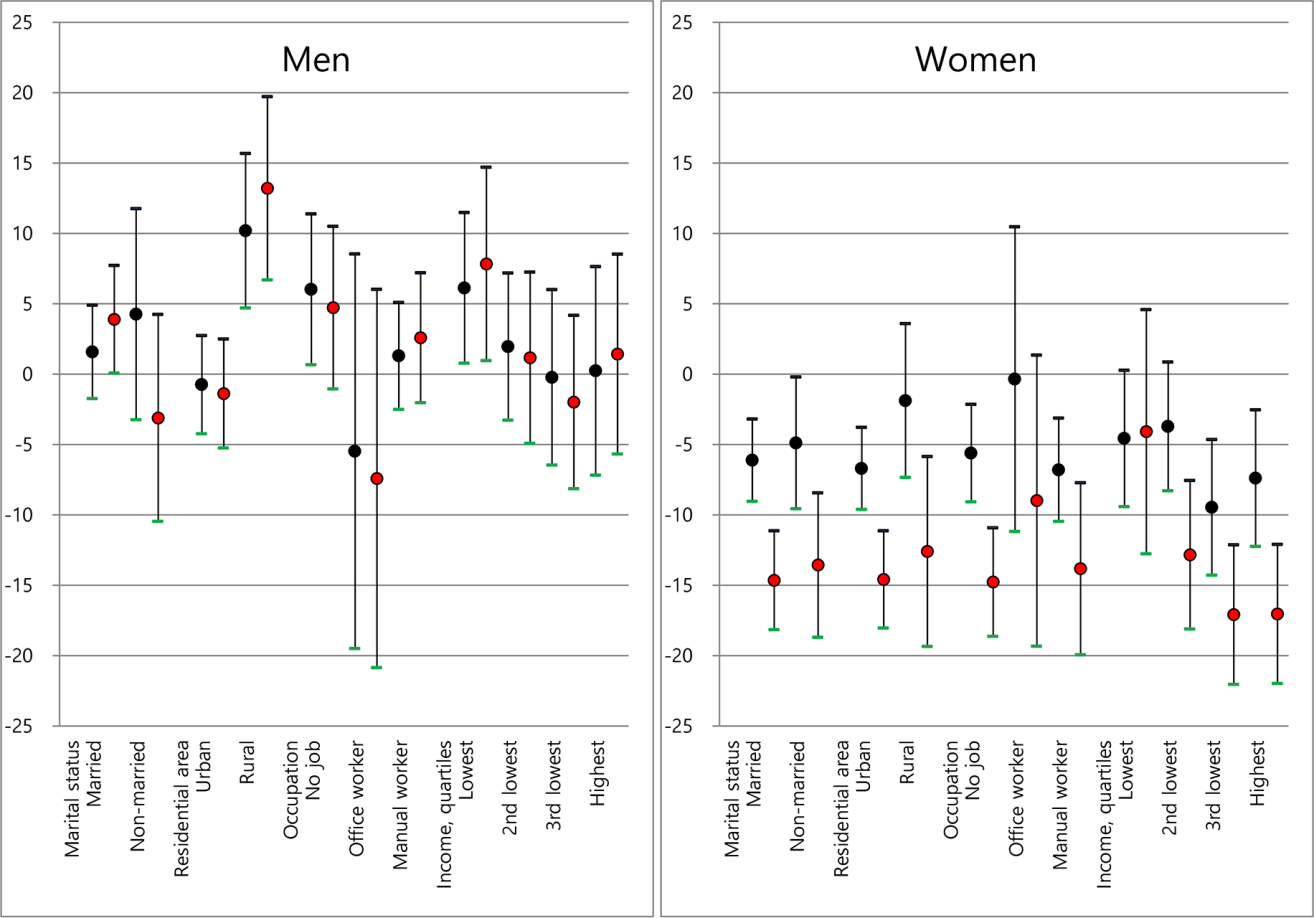
The percentage change in an individual’s predicted probability of being obese (its 95% CIs), if the individual belonging to a socioeconomic status indicator group would increase the individual’s level.

Fig 1 shows the change in an individual’s predicted probability of being obese (percentage point), if the socioeconomic status indicator group they belonged to were to increase their education from a lower level (middle school or less) to a higher level (either high school; or college or higher) with all the other factors held constant at each individual’s own values. In men, for most socioeconomic status indicator groups, an increase in education seemed to show no significant change in obesity risk. However, for the following socioeconomic status indicator groups, obesity risk appeared to increase significantly owing to an increase in their education level: 1) in the married group, the predicted probability of being obese significantly increased by 7.7% from middle school or less to college or higher (*p* = 0.045); 2) the rural resident group demonstrated an increase of 15.7% to high school (*p* < 0.001) and a 19.7% to college or higher (*p* < 0.001); 3) for the group who had no job, an 11.4% increase to high school (*p* = 0.028); and 4) for the lowest income group, an 11.5% increase to high school (*p* = 0.025) and 14.7% increase to college or higher (*p* = 0.025) was observed.

Meanwhile, in women, education seemed to have a negative association with obesity risk for most of the socioeconomic status indicator groups, i.e., the predicted probability of being obese decreased significantly from the lowest level (middle school or less) to a higher level. For example, for the married group, the predicted probability of being obese showed an 11.1% decrease to college or higher (*p* < 0.001), and for the highest income group, a 12.1% decrease to college or higher (*p* < 0.001). However, for the following socioeconomic status indicator groups, an increase in education from middle school or less to a higher level showed no significant association with obesity risk: 1) for the rural resident group, the predicted probability of being obese had no significant change when their education level increased to high school; 2) for the office worker group, when their education level increased both to high school and to college or higher; 3) for the lowest income group, when their education level increased both to high school and to college or higher; and 4) for the second lowest income group, when their education level increased to high school. These results suggest that the role of education on the association between a socioeconomic status indicator and obesity may differ depending on both the type of the socioeconomic status indicator and sex under investigation.

## Discussion

In this study, we investigated the role of education on the association of each socioeconomic status indicator with obesity. From the results obtained from all methods mentioned previously, we discovered that education might or might not play a role on the association of each socioeconomic status indicator with obesity, depending on the socioeconomic status indicator and sex under consideration. For example, the results from logistic regression models after adjustments for all the studied confounders provides interesting suggestions: in men, education may be neither a confounder nor an effect modifier in the associations between occupation and obesity as well as between income and obesity; whereas, education may be an effect modifier in the association between marital status and obesity and function both as a confounder and an effect modifier in the associations between residential area and obesity. In contrast, in women, education may be neither a confounder nor an effect modifier in the association between marital status and obesity as well as between occupation and obesity, whereas education may be a confounder in the association between residential area and obesity and both a confounder and an effect modifier in the associations between income and obesity.

This study also suggests that because the role of education on the association between each socioeconomic status indicator and obesity differs according to the socioeconomic status indicator and sex under investigation, education may be either negatively or positively associated with obesity risk according to the socioeconomic status indicator and sex under investigation. This study found that an enhanced education might be associated with a higher risk of obesity in men for the following groups like the married group, the rural resident group, the unemployed group, and for the lowest income group, in sharp contrast that in women, education may have a negative association with obesity in most groups of all socioeconomic status indicators.

With regard to the relationship between socioeconomic status indicators and obesity in developed countries, previous studies without considering interaction effects of such indicators reported a so-called “inverse association between socioeconomic status and obesity risk,” stating that socioeconomic status is negatively associated with obesity risk in both men and women [4–6,22]. However, after considering the role of education on the associations of other socioeconomic status indicators with obesity risk, this study found no evidence of a negative association of education with obesity risk in men.

Some recent studies of developed countries shed doubt on the perceived inverse association. These studies suggest that the direction of the associations between socioeconomic status indicators and obesity risk may differ by sex and that the associations may not be significant for a specific sex. Further, the type of socioeconomic status indicator associated with obesity risk may also vary by sex. In Canada, the association between income and obesity risk was significant both in men and in women, but the direction of the association was sharply contrasted by sex; obesity risk was higher in rich men, but obesity risk was higher in poor women [7]. In France, obesity risk was associated with occupation in men, whereas it was associated with educational level and frequency of holiday trips in women [8]. In Luxembourg, education was significantly associated with obesity in women, but not in men [9]. In the United States, education had no significant association with obesity in men, but in women, those with college degrees had a higher likelihood of being obese than their less educated counterparts [10]. In South Korea, both income and education showed no association with obesity in men, whereas education, not income, was inversely associated with obesity in women [11].

To date, no study of obesity risk for people in developed countries has assessed, in detail, the role of education on the associations between other socioeconomic status indicators and obesity. It is surprising that in developing countries, the role of education as a socioeconomic status indicator linked to obesity has been examined in some studies, although they were only for women of limited age without considering different socioeconomic status indicators. In Egypt, for women of reproductive age, education reduced obesity risk in its interplay with wealth [23]. In China, education interacted with occupation in regards to abdominal obesity of women at least 60 years old. In women with no education, individuals with a sedentary occupation were more likely to be obese than those with an agricultural occupation. However, there was no difference in the likelihood between occupational groups in women with any education [24].

Meanwhile, we will discuss plausible mechanisms that may explain the two important findings in this study. The first important finding is that education may play a role as either a confounder or an effect modifier in the association of another socioeconomic status indicator with obesity risk. The reasons for this may be partly attributed to how education and another socioeconomic status indicator determine each other, thereby influencing obesity risk in a combined manner. Particularly, in the case of this study, which analyzed an adult population aged ≥25 years that had most likely completed their education, education is more likely to influence another socioeconomic status indicator, rather than the socioeconomic status indicator influencing education level. Many studies from different disciplines document significant evidence regarding the effects of education level on marital status, residential area, occupation, and income [25–30].

The second important finding in this study is that the role of education on the associations between another socioeconomic status indicator and obesity risk differs by sex. These differences by sex may result from sex differences in knowledge on certain choice of diets, nutrition and nutritional beliefs through education, as implied in a study of college students in the United States [31]. In addition, as shown in previous studies examining the main effects of socioeconomic status indicators on obesity risk [7–11], the association of each socioeconomic status indicator including education with obesity risk may differ by sex, and the effect of education on other socioeconomic status indicators may also differ by sex [25–30]. In addition, there may be different socioeconomic circumstances experienced by men and women, although it may be difficult to sufficiently control for these circumstances in most empirical models. For example, even in developed countries, in order to marry a desirable partner, a woman with a college degree and working in an office may take greater efforts to avoid appearing obese than her male counterpart. Various literature documents existing sex differences involving an obesity penalty in employment settings, in health-care settings, in educational settings, in interpersonal relationships and in marriage settings [32–37]. In relation to this, it needs to be noted that although South Korea ranked 11^th^ in the size of national economy in 2015 according to the World Bank [12], ironically, it ranked 115^th^ in the global gender gap index according to the World Economic Forum [38]. Therefore, it is not difficult to find evidence of pronounced sex discrimination against women in South Korea [39–42], hence highly educated women in South Korea seem to be at a higher risk towards the obesity penalty than that of their counterparts in other developed countries.

As far as we know, this is the first multi-dimensional study to investigate the role of education on the associations between other socioeconomic status indicators and obesity in a developed country. Though caution must be exercised in drawing policy suggestions from cross-sectional data results, this study suggests that depending on sex, increased education may raise obesity risk or have no effect on it through its interplay with other socioeconomic characteristics. These results could raise a question whether or not an enhanced education is an efficient policy tool to achieve a goal of health attainment for a certain population group like the reduction of obesity risk for adult men in South Korea, as discussed in previous studies [43–45].

This study analyzed data from the most recent sample of nationally representative adults in South Korea that included rich information about anthropometric measures, demographic characteristics, socioeconomic status, health behaviors, dietary intake, psychological characteristics, and diagnosed diseases. Most advantageously, this study explored the role of education on the associations between various other indicators of socioeconomic status and obesity in developed countries.

This study has several limitations. First, because this was a cross-sectional study, we could not draw a causal relationship between education, other socioeconomic status indicators and obesity. If we had obtained cohort data, we could have included time-varying covariates in our statistical analysis. Second, self-reporting methods for some information may have caused recall bias and measurement error. Third, other potential covariates, such as quality of education, genetics, peer effects, diet quality, and parental obesity, could not be considered because of lack of information. Fourth, this study examined the interactions on the multiplicative scale because it did not aim to examine if the interactions were either on an additive scale or on a multiplicative scale. As shown in most published epidemiological studies, interactions have been reported on the multiplicative scale [46,47]. However, we would like to note that the presence and direction of interaction on the additive scale is important for public health relevance [48]. Fifth, we failed to draw directed acyclic graphs (DAGs) in this study, because it was very difficult to draw them from the models with all the studied, socioeconomic status indicators, as well as all the studied, potential confounders. We fully understand that DAGs would be very useful when researchers try to explain confounding and effect modification between exposure and outcome, as a respected, anonymous reviewer has commented [49]. Finally, unobserved factors, such as time preference and risk aversion, may have influenced both socioeconomic status and obesity [50,51].

## Conclusions

The results of this study suggests role of education on the association between other socioeconomic status indicators and obesity risk, which is influenced by sex. This should be considered in policy efforts to reduce obesity risk in South Korea. Future research is needed to examine whether these results are valid in other settings in terms of either socio-culture or economic development.
